# Immediate Death: Not So Bad If You Discount the Future but Still Worse than It Should Be

**DOI:** 10.1177/0272989X251325828

**Published:** 2025-03-20

**Authors:** Eleanor M. Pullenayegum, Marcel F. Jonker, Henry Bailey, Bram Roudijk

**Affiliations:** Child Health Evaluative Sciences, The Hospital for Sick Children, Toronto, ON, Canada; Dalla Lana School of Public Health, University of Toronto, Toronto, Canada; Erasmus School of Health Policy & Management, Erasmus Centre for Health Economics and Erasmus Choice Modelling Centre, Erasmus University Rotterdam, The Netherlands; Department of Economics & HEU Centre for Health Economics, the University of the West Indies, St Augustine, Trinidad, West Indies; EuroQol Research Foundation, Rotterdam, The Netherlands

**Keywords:** health state valuation, discrete choice experiment

## Abstract

**Objectives:**

Discrete choice experiments (DCEs) as a valuation method require preferences to be anchored on the quality-adjusted life-year scale, usually through tasks involving choices between immediate death and various impaired health states or between health states with varying durations of life. We sought to determine which anchoring approach aligns best with the composite time tradeoff (cTTO) method, with a view to informing a valuation protocol that uses DCEs in place of the cTTO.

**Methods:**

A total of 970 respondents from Trinidad and Tobago completed a DCE with duration survey. Tasks involved choosing between 2 lives with identical durations, followed by a third option, representing either full health for a number of years or immediate death. Data were analyzed using mixed logit models, both with and without exponential discounting for time preferences.

**Results:**

Assuming linear time preferences, the estimated utility of immediate death was −2.1 (95% credible interval [CrI] −3.2 to −1.2) versus −0.28 (95% CrI −0.47, −0.10) when allowing for nonlinear time preferences. Under linear time preferences, the predicted health-state values anchored on duration had range (−1.03, 1) versus (0.34, 1) when anchored on immediate death. The ranges under nonlinear time preferences were (−0.54, 1) versus (−0.22, 1). The estimated discount parameter was 23% (95% CrI 22% to 25%).

**Conclusions:**

The nonzero discount parameter indicates that time preferences were nonlinear. Nonlinear time preferences anchored on duration provided the closest match to the benchmark EQ-VT cTTO values in Trinidad and Tobago, whose range was (−0.6, 1). Thus, DCE with duration can provide similar values to cTTO provided that nonlinear time preferences are accounted for and anchoring is based on duration.

**Highlights:**

- Time preferences for health states in Trinidad and Tobago were nonlinear.
- In discrete choice tasks, we show that immediate death has a utility less than zero.
- DCE utilities under nonlinear time preferences with anchoring on duration agreed well with cTTO utilities.

Quality-adjusted life-years are a key component in economic evaluations in many countries^1–4^ and typically rely on instruments such as the EQ-5D-5L,^5^ SF-6D,^6^ or HUI3^7^ to elicit health utilities. These instruments require a value set that specifies the population utility associated with each health state captured by the instrument. Discrete choice experiments (DCEs) have emerged as a promising approach to creating such value sets, as they can be done online and do not need to be administered by an interviewer.^8,9^ This makes them an attractive alternative to the widely used composite time tradeoff (cTTO),^10^ which needs to be administered by a trained interviewer. It is thus of interest to examine whether DCEs can yield similar results to cTTO tasks: if this is the case, then costly interviewer-administered cTTO protocols could be replaced by DCEs.

When respondents choose between 2 health states without duration, preferences can be inferred on a latent scale (i.e., up to a linear transform). Some additional information is required to anchor the utilities on the full-health–dead scale (where full health has a utility of 1 and being dead has a utility of 0). These anchors can be either external^11,12^ or based on additional discrete choice tasks. This latter option is the focus of this article. There are 2 types of additional tasks that can be used to anchor the latent utilities: a discrete form of TTO anchoring that involves trading off an impaired health state of a specified duration with full health for a shorter duration or trading off impaired health states of a specified duration with immediate death.

Under a QALY model, both being dead for any duration and a health state of duration zero should have a utility of zero; see Roudijk et al.^13^ for a detailed theoretical justification. Several studies have assumed that immediate death also has a utility of zero^8,9^; however, there is empirical evidence that this is not the case and that its utility is lower than zero.^14,15^ This finding is consistent with qualitative findings from TTO interviews, which suggest that there is both a discontinuity in preferences as durations approach zero^16,17^ as well as heterogeneity in how people interpret immediate death.

In dealing with either of these issues, a third issue must be contended with: time preferences are nonlinear. There is empirical evidence that respondents discount future health status in favor of improved health now.^18,19^ However, estimating this discount rate requires careful selection of discrete choice tasks to make the parameter identifiable.^20^

An important limitation in previous work exploring anchoring on immediate death is that the DCE tasks were not designed to permit estimation of the discount parameter. It is therefore currently unknown whether anchoring latent utilities from DCEs on immediate death remains problematic when incorporating discounting into the estimation procedure.

Two valuation studies of the EQ-5D-5L in Trinidad and Tobago were recently published. The first valuation study used the international EQ-5D-5L EQ-VT valuation protocol based on cTTO tasks,^10^ whereas the second used DCE with duration following a protocol^19,21,22^ that permits the estimation of nonlinear time preferences and compared the results with that those obtained using the EQ-VT valuation protocol.^23^

In this work, we use data collected in the Trinidad and Tobago DCE with a duration valuation study to examine whether immediate death continues to have a lower utility than a state of duration zero after accounting for discounting. We compute value sets anchoring on a duration of zero and anchoring on immediate death and examine how well the range of the value sets agrees with the range of the value set based on cTTO,^23^ to inform recommendations on how to anchor latent scale DCE utilities.

## Methods

### Population

This study is a secondary analysis of an existing sample of 970 respondents included in the Trinidad and Tobago DCE with duration study.^24^ This study used quota sampling to achieve a population that was representative of the general population in terms of age, sex, and geography. Recruitment was through a panel company, which used both an internet panel (e-mailed links to the survey) and recruitment in public places (e.g., libraries, transit hubs) with survey completion done on the recruiter’s laptop. Ethical approval was obtained from the University of The West Indies (exemption letter CREC-SA.1468/03/2022 dated March 7, 2022).

### Task Types

Each respondent completed 1 set of 18 split triplets,^25^ 15 of which involved tradeoffs with full health and 3 of which involved tradeoffs with immediate death. Each triplet began with a pair of health states of equal duration (life A and life B), from which the respondent was asked to choose their preference. To simplify the task, life A and life B differed in just 3 of the 5 EQ-5D-5L dimensions; the other 2 dimensions were the same in life A and life B.

Regardless of the stated preference, in the second half of the triplet, life A was blurred and the respondent was asked to choose between life B and life C. In those split triplets involving tradeoffs with full health, life C was defined as full health but with a shorter duration than life B; this choice is thus a discrete version of a traditional TTO task. In those split triplets involving tradeoffs with immediate death, life C was immediate death.

### DCE Design

A near-orthogonal design was used initially, the responses to which (*n* = 211) were analyzed to create a more efficient design using the TPC-QD software package.^20^ The design was further updated at intervals of 200 respondents until the priors used to generate the design did not change substantially between updates. Durations were whole years from 1 up to and including 15, with an additional duration of 6 mo. Each design contained 10 subdesigns with 18 split triplets as described above. Respondents were randomly assigned to 1 of the 18 split triplets, with the order of lives A and B also randomly assigned.

### Analytic Plan

#### Utility model

We assumed a main effects model and subject-specific regression coefficients for the latent utilities to account for between-subject heterogeneity in preferences. Specifically, letting $U_{\mathrm{ijt}}$ be the latent utility for respondent *i* valuing health state *j* with a duration *t*, we used a mixed logit model with discounting, that is,

$$U_{\mathrm{ijt}}=X_{j}\beta_{i}D\left(t;\rho \right)+\beta_{\mathrm{ip}+1}I\left(\mathrm{state}\;j\;\mathrm{is}\;\mathrm{immediate}\;\mathrm{death}\right)+\sigma \epsilon_{\mathrm{ijt}}$$

where *X_j_* is a *p*-dimensional row vector of attributes of health state *j* whose first element is 1 to provide an intercept, $\epsilon_{ijt}\sim$ iid Gumbel, $\beta_{i}\sim \mathrm{MVN}(\beta ,\Sigma_{\beta })$ , I() denotes an indicator function, and D(t;ρ ) is the discounted duration under exponential discounting with discount parameter ρ, that is, $D\left(t;\rho \right)=\;\frac{1-\exp\left(\rho t\right)}{\exp\left\{\rho \right\}-1}$ for ρ > 0 and *D*(*t*; ρ) = *t* for ρ = 0.

We assumed a main effects functional form for the design matrix, specifically *X_j_* = (1, MO2_j_, MO3_j_, MO4_j_, MO5_j_, SC2_j_, SC3_j_, SC4_j_, SC5_j_, UA2_j_, UA3_j_, UA4_j_, UA5_j_, PD2_j_, PD3_j_, PD4_j_, PD5_j_, AD2_j_, AD3_j_, AD4_j_, AD5_j_), where MO2_j_, MO3_j_, MO4_j_, MO5_j_ are indicators (0 = no, 1 = yes) for whether mobility in health state *j* is at level 2, 3, 4, or 5, respectively, and similarly for self-care, usual activities, pain/discomfort, and anxiety/depression.

#### Discount parameter

We fitted 2 models. The first assumed no discounting, that is, ρ = 0, whereas the second used a uniform(0,1) prior for ρ.

#### Estimation

Models were estimated using OpenBugs, using 3 chains, a burn-in of 50,000 and 50,000 draws from the posterior distributions. Convergence was evaluated based on inspection of the chains and diagnostics proposed by Geweke.^26^ The BUGS models, including the exact specification of the prior distributions, are included in the online supplemental.

#### Anchoring

The fitted models do not estimate the coefficients β but rather scaled versions that must be anchored. Specifically, the fitted models yield estimates of $\Sigma_{\beta }/\surd 2\sigma$ , $\beta^{*}=\beta /\surd 2\sigma$ , and ρ. To anchor the utilities to the full health–dead scale, we have 2 options:

Option 1 is to assume that immediate death has a utility of zero. Since full health has a utility of 1, the difference in utility between full health and immediate death is 1. Referring back to equation (1) for the anchored coefficients, we have $\beta_{1}-\beta_{p+1}=1$ ; it then follows that $\beta_{1}^{*}-\beta_{p+1}^{*}\;=\;1/\sqrt{2}\sigma$ so that $\beta =\beta^{*}/(\beta_{1}^{*}-\;\beta_{p+1}^{*})$ .
Option 2 is to anchor on duration and note that since full health has a utility of 1 by definition, we have $\beta_{1}$ = 1, and thus, $\beta_{1}^{*}=\;1/\surd 2\sigma$ so $\beta =\;\beta^{*}/\beta_{1}^{*}.$

We anchored the utilities using each option in turn.

## Results

As can be seen from Table 1, both the choice of anchor and the choice of time preferences affect the coefficients.

**Table 1:** Disutilities (Standard Errors) under Linear and Nonlinear Time Preferences and with Anchoring on Either Immediate Death or Duration

	Linear Time Preferences		Nonlinear Time Preferences		cTTO
	Anchored on		Anchored on		
	Immediate Death	Duration	Immediate Death	Duration	
Mobility level 2	0.021 (0.015, 0.028)	0.065 (0.047, 0.088)	0.025 (0.015, 0.036)	0.032 (0.019, 0.046)	0.014 (0.000, 0.028)
Mobility level 3	0.048 (0.038, 0.060)	0.146 (0.115, 0.188)	0.075 (0.061, 0.090)	0.095 (0.078, 0.113)	0.058 (0.033, 0.083)
Mobility level 4	0.095 (0.079, 0.114)	0.29 (0.232, 0.368)	0.169 (0.147, 0.193)	0.215 (0.191, 0.240)	0.162 (0.135, 0.189)
Mobility level 5	0.153 (0.128, 0.187)	0.469 (0.384, 0.589)	0.284 (0.250, 0.322)	0.361 (0.328, 0.397)	0.357 (0.330, 0.384)
Self-care level 2	0.026 (0.020, 0.034)	0.080 (0.059, 0.108)	0.031 (0.020, 0.042)	0.039 (0.025, 0.054)	0.029 (0.015, 0.043)
Self-care level 3	0.037 (0.030, 0.047)	0.115 (0.088, 0.151)	0.057 (0.044, 0.070)	0.072 (0.056, 0.088)	0.074 (0.052, 0.096)
Self-care level 4	0.084 (0.070, 0.102)	0.256 (0.205, 0.324)	0.142 (0.124, 0.163)	0.181 (0.160, 0.203)	0.159 (0.134, 0.184)
Self-care level 5	0.121 (0.102, 0.145)	0.369 (0.295, 0.467)	0.223 (0.196, 0.253)	0.283 (0.257, 0.313)	0.221 (0.197, 0.245)
Usual activities level 2	0.023 (0.015, 0.033)	0.069 (0.050, 0.090)	0.017 (0.006, 0.028)	0.022 (0.007, 0.036)	0.016 (0.002, 0.030)
Usual activities level 3	0.037 (0.027, 0.049)	0.111 (0.089, 0.142)	0.045 (0.032, 0.059)	0.058 (0.041, 0.074)	0.087 (0.065, 0.109)
Usual activities level 4	0.069 (0.056, 0.087)	0.210 (0.175, 0.262)	0.107 (0.090, 0.124)	0.135 (0.116, 0.156)	0.145 (0.123, 0.167)
Usual activities level 5	0.098 (0.081, 0.123)	0.300 (0.251, 0.374)	0.166 (0.144, 0.189)	0.210 (0.187, 0.236)	0.216 (0.191, 0.241)
Pain/discomfort level 2	0.029 (0.022, 0.037)	0.089 (0.064, 0.118)	0.046 (0.035, 0.059)	0.059 (0.045, 0.073)	0.026 (0.014, 0.038)
Pain/discomfort level 3	0.046 (0.037, 0.057)	0.140 (0.110, 0.181)	0.078 (0.064, 0.093)	0.099 (0.083, 0.116)	0.102 (0.077, 0.127)
Pain/discomfort level 4	0.101 (0.083, 0.122)	0.308 (0.246, 0.390)	0.181 (0.157, 0.209)	0.230 (0.205, 0.258)	0.316 (0.291, 0.341)
Pain/discomfort level 5	0.153 (0.128, 0.186)	0.469 (0.378, 0.591)	0.287 (0.250, 0.328)	0.365 (0.329, 0.403)	0.541 (0.510, 0.572)
Anxiety/depression level 2	0.030 (0.023, 0.038)	0.093 (0.071, 0.122)	0.042 (0.031, 0.053)	0.053 (0.039, 0.067)	0.024 (0.012, 0.036)
Anxiety/depression level 3	-0.060 (0.050, 0.072)	0.184 (0.140, 0.238)	0.100 (0.084, 0.117)	0.127 (0.109, 0.146)	0.057 (0.033, 0.081)
Anxiety/depression level 4	0.114 (0.096, 0.136)	0.349 (0.274, 0.446)	0.205 (0.179, 0.234)	0.261 (0.233, 0.291)	0.168 (0.144, 0.192)
Anxiety/depression level 5	0.136 (0.115, 0.162)	0.418 (0.328, 0.533)	0.255 (0.224, 0.290)	0.324 (0.292, 0.360)	0.272 (0.250, 0.294)
Immediate death	n/a	2.102 (1.159, 3.210)	n/a	0.275 (0.099, 0.469)	n/a

Time preferences were not linear; the estimated discount rate parameter has a posterior mean of 23.4% with a 95% credible interval (CrI) of 21.7% to 25.1%. Assuming linear time preferences (i.e., fixing the discount parameter at zero) generally led to smaller disutilities when anchoring on immediate death, while it led to larger disutilities when anchoring on a duration of zero (Table 1).

Anchoring on immediate death led to smaller disutilities than anchoring on zero duration regardless of whether or not time preferences were assumed to be linear, although the effect was more pronounced under linear time preferences. See Table 1 for tabulated regression coefficients.

Furthermore, when time preferences were assumed to be linear and anchoring was on duration, immediate death had a posterior mean utility of −2.1 (95% CrI −3.2 to −1.2). This increased substantially to −0.28 (95% CrI −0.47, −0.10) on allowing for nonlinear time preferences (Table 1).

**Figure 1:**
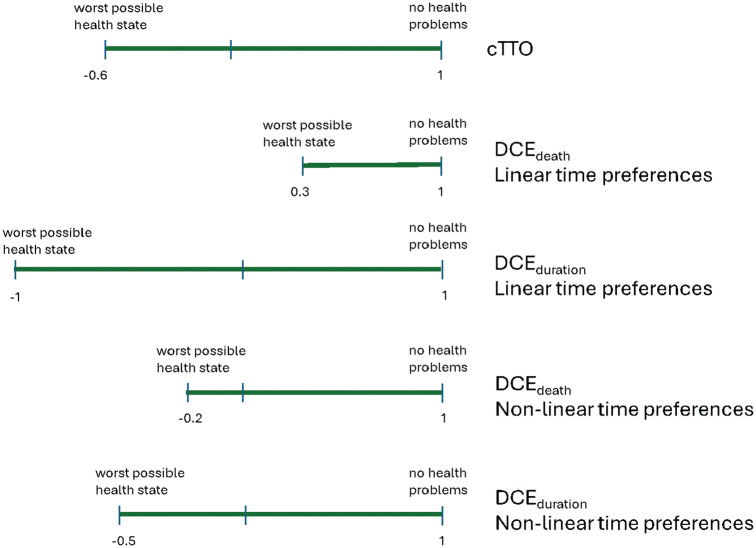
Length of the quality-adjusted life-year (QALY) scale under different time preferences and anchor choices.

**Figure 2:**
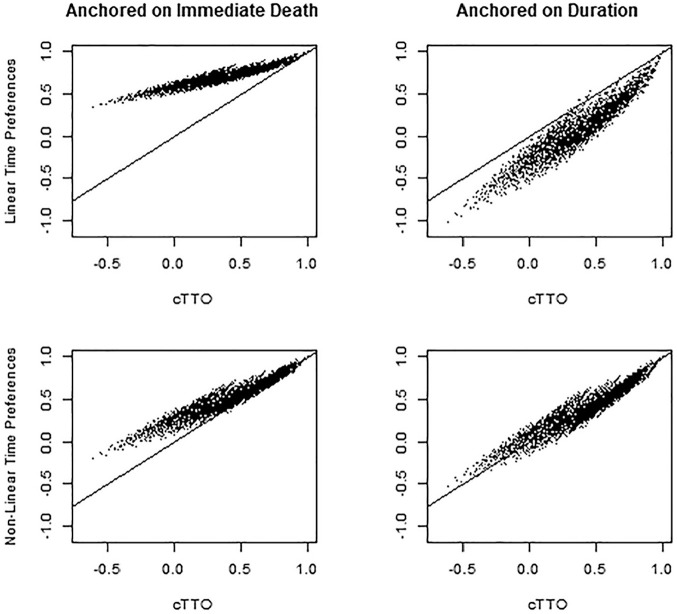
Comparison of discrete choice experiment (DCE)–based tariffs to the composite time tradeoff (cTTO) tariff.

The worst health state had an estimated utility of 0.34 (95% CrI 0.20, 0.44) with linear time preferences anchored on immediate death, −1.03 (95% CrI −1.54, −0.65) with linear time preferences anchored in duration, −0.21 (95% CrI −0.37, −0.08) with nonlinear time preferences anchored on immediate death, and −0.54 (95% CrI −0.69, −0.41) with nonlinear time preferences anchored on duration (Figure 1). For comparison, the reported utility for the worst health state using cTTO was −0.61. Correspondence between the health state utilities under cTTO and DCE are shown in Figure 2, with nonlinear time preferences anchored on duration providing the closest correspondence.

## Discussion

A unique contribution of this article is that we have compared anchoring on duration versus anchoring on immediate death while accounting for nonlinear time preferences. Our findings in a general population sample from Trinidad and Tobago are that, first, immediate death does not have a utility of zero (this is true regardless of whether linear time preferences are assumed, although the utility is closer to zero on assuming nonlinear time preferences); second, that time preferences are nonlinear; and third, that when comparing the 4 DCE QALY tariffs with the cTTO tariff, assuming nonlinear time preferences and anchoring on duration yields close agreement, whereas the other 4 choices yield poor agreement.

Anchoring on immediate death resulted in utilities estimated through DCEs exceeding the cTTO utilities. Since the mixed logit attributes a coefficient for immediate death that is less than zero, when the utility scale is anchored on immediate death, a zero duration will be given a utility that is greater than 0 (since immediate death is placed at zero, a duration of zero is positioned above zero), and therefore, the preferences for the EQ-5D-5L health states are shifted toward 1. Anchoring on a duration of zero and using linear time preferences led to DCE utilities that were in most cases lower than cTTO utilities. It is unclear why this is the case; what is clear is that failing to take the curvature in the time preferences into account when determining the anchor point for zero duration results in too many health states valued below 0.

The nonlinear time preferences we observed have also been noted in a number of valuation studies using both DCE^18,19,27^ and TTO,^28–30^ and a greater impact for DCE-based valuation over TTO-based valuation has been hypothesized.^19^ Mistakenly assuming linear time preferences led to a utility scale ranging from 0.338 to 1 when anchoring on immediate death or to a range of −1.026 to 1 when anchoring on a duration of zero (with immediate death having an estimated utility of −2.1). The utility range on anchoring on immediate death, and the utility attached to immediate death are, in our opinion, unreasonable. Thus, the assumption of linear time preferences is not only empirically refuted by the estimated discount parameter having a posterior distribution with most of its mass away from zero but also leads to a value set that lacks face validity. This has important implications for the design of DCE studies incorporating duration: specifically, the choice tasks need to be selected in such a way as to make the discount parameter identifiable. When using a D-efficient design, this corresponds to selecting an analytic model that includes nonlinear time preferences and using design updates as data accumulates to optimize information about the discount parameter; see Jonker and Bliemer^20^ for details on how to achieve this.

A shifting of preferences for immediate death away from zero on anchoring the tariff using duration has also been noted elsewhere. For example, immediate death was reported to have utilities of −0.46 (95% CI −0.79, −0.02) and −3.94 (−5.56, −2.36) in Australian studies of the EQ-5D-5L and SF-6D, respectively, on using the mixed logit model.^15^ Under a conditional logit model, anchoring on immediate death has been noted to lead to a shorter scale than anchoring on full health.^31^ Notably, however, these analyses all assumed linear time preferences.

There are several explanations for the shift of immediate death away from zero. While equivalence to death has been formally defined,^32^ the processes by which respondents decide whether something is better or worse than dead do not always match this definition^33^ and are, moreover, sensitive to framing.^34^

We have studied the EQ-5D-5L in Trinidad and Tobago; generalizability to other countries and to other instruments needs to be carefully considered. Preferences have been noted to be country specific, and in particular, values around immediate death and life span in impaired health states may vary across cultures. We see no reason why the results would not generalize to other instruments such as the QLU-C10,^35^ FACT,^36^ or SF-6D,^37^ for which valuation has been done using DCE with duration, and indeed nonlinear time preferences have been noted for the WOOP and SF-6D^19,21^ and a reduced utility range on anchoring on immediate death has been noted for the SF-6D.^38^

We are fairly confident about the quality of the cTTO data as this used a protocol with an established quality-control procedure^10^; however, this was not the case for the DCE with duration data. While we excluded speeders (see Roudijk et al.^24^ for further details), there was no interview debrief, and hence, we have no information on whether respondents either understood or engaged with the task, beyond noting that the estimated utility decrements show good face validity and that population-level preferences for the cTTO align with those for the DCE with duration data when modeled and anchored appropriately.

While we have shown that nonlinear preferences anchored on duration align well with cTTO preferences at the population level, we have not shown agreement at the individual level. This is not possible to evaluate in our data as the DCE with duration and cTTO tasks were completed by different respondents.

Given the lack of face validity on anchoring on immediate death and previously described theoretical concerns over choices involving death,^39^ investigators using DCE for health state valuation may wish to consider dropping tasks involving comparisons to immediate death and instead consider comparisons with full health.

When nonlinear time preferences were accounted for and when anchoring was on duration, the observed utility range of −0.55 to 1 agreed well with that obtained for Trinidad and Tobago^23^ using cTTO preferences elicited using the widely used EQ-VTv2 protocol^10^ (utilities ranged from −0.6 to 1 ). Moreover, the 2 sets of preferences agreed well not just in range but at the individual state level.^24^

In summary, we recommend that valuation studies using DCEs with duration design the choice tasks so as to be able estimate discount parameters and examine whether nonlinear time preferences are present. We further suggest that, given respondents’ potential for heterogeneous interpretations of immediate death, tariffs be anchored on duration rather than immediate death.

## Supplemental Material

sj-docx-1-mdm-10.1177_0272989X251325828 – Supplemental material for Immediate Death: Not So Bad If You Discount the Future but Still Worse than It Should Be Supplemental material, sj-docx-1-mdm-10.1177_0272989X251325828 for Immediate Death: Not So Bad If You Discount the Future but Still Worse than It Should Be by Eleanor M. Pullenayegum, Marcel F. Jonker, Henry Bailey and Bram Roudijk in Medical Decision Making
