# Supplementary material for: Immediate Death: Not So Bad If You Discount the Future but Still Worse than It Should Be
Source: Med Decis Making. 2025 Mar 20;45(4):376–84. doi: 10.1177/0272989X251325828 (PMC11992645; doi:10.1177/0272989X251325828)
Supplement: sj-docx-1-mdm-10.1177_0272989X251325828 – Supplemental material for Immediate Death: Not So Bad If You Discount the Future but Still Worse than It Should Be [file sj-docx-1-mdm-10.1177_0272989X251325828.docx]

**Immediate death: not so bad if you discount the future, but still worse than it should be.**

**Running title: Immediate death: worse than it should be**

**Appendix:**

**Model code linear model**

model {

# N = number of respondents

# T = number of choice tasks per respondent

# A = number of alternatives per choice task

# V = number of explanatory variables (including non-linear time preference)

# likelihood

for (n in 1:N){

for (t in 1:T){

Y[n,t] <- 1

Y[n,t] ~ dcat(prob[n, t, 1:2])

}}

# prob calculations <- user-written softmax function

for (n in 1:N){

for (t in 1:T){

prob[n,t,1:2] <- softmaxExpDeath(X[n,t,1,], Q[n,t,1], X[n,t,2,], Q[n,t,2], beta[n,], rate)

}}

# priors

# multivariate normal prior on beta

for (n in 1:N){ beta[n,1:V] ~ dmnorm(mu_beta[], prec_beta[,]) }

mu_beta[1:V] ~ dmnorm(hyper_mu_beta[],hyper_tau_beta[,])

prec_beta[1:V,1:V] ~ dwish(scaleMatrix[,],V)

for (b in 1:V){

hyper_mu_beta[b] <- 0

for (bb in 1:V){

scaleMatrix[b,bb] <- equals(b,bb)

hyper_tau_beta[b,bb] <- equals(b,bb)/100

}}

# normal prior on discount rate

rate <-0.0

# additional computations

# population SD

covar[1:V,1:V] <- inverse(prec_beta[,])

for (v in 1:V){ SD[v] <- sqrt(covar[v,v]) }

# log-likelihood

for (n in 1:N){

for (t in 1:T) { LL_task[n,t] <- log( prob[n,t, Y[n,t] ]) }

LL_resp[n] <- sum(LL_task[n,])

}

LL <- sum(LL_resp[])

# McFadden R-squared

LL_random <- N*T*log(0.5)

Rsq <- (LL - LL_random)/-LL_random

# QALY estimates (duration)

QALY_DUR[1] <- 1

for (v in 2:V){

QALY_DUR[v] <- mu_beta[v] / mu_beta[1]

}

#worst possible health state 55555

worst[1] <- QALY_DUR[1] + QALY_DUR[5] + QALY_DUR[9] +QALY_DUR[13] +QALY_DUR[17] + QALY_DUR[21]

# QALY estimates (immediate death)

QALY_DEAD[1] <- 1

for (v in 2:V-1){

QALY_DEAD[v] <- QALY_DUR[v] * (1/(1-QALY_DUR[V]) )

}

QALY_DEAD[V] <-0

worst[2] <- QALY_DEAD[1] + QALY_DEAD[5] + QALY_DEAD[9] +QALY_DEAD[13] +QALY_DEAD[17] + QALY_DEAD[21]

}

}

**Model code non-linear model**

model {

# N = number of respondents

# T = number of choice tasks per respondent

# A = number of alternatives per choice task

# V = number of explanatory variables (including non-linear time preference)

# likelihood

for (n in 1:N){

for (t in 1:T){

Y[n,t] <- 1

Y[n,t] ~ dcat(prob[n, t, 1:2])

}}

# prob calculations <- user-written softmax function

for (n in 1:N){

for (t in 1:T){

prob[n,t,1:2] <- softmaxExpDeath(X[n,t,1,], Q[n,t,1], X[n,t,2,], Q[n,t,2], beta[n,], rate)

}}

# priors

# multivariate normal prior on beta

for (n in 1:N){ beta[n,1:V] ~ dmnorm(mu_beta[], prec_beta[,]) }

mu_beta[1:V] ~ dmnorm(hyper_mu_beta[],hyper_tau_beta[,])

prec_beta[1:V,1:V] ~ dwish(scaleMatrix[,],V)

for (b in 1:V){

hyper_mu_beta[b] <- 0

for (bb in 1:V){

scaleMatrix[b,bb] <- equals(b,bb)

hyper_tau_beta[b,bb] <- equals(b,bb)/100

}}

# normal prior on discount rate

rate ~ dunif(0,1)

# additional computations

# population SD

covar[1:V,1:V] <- inverse(prec_beta[,])

for (v in 1:V){ SD[v] <- sqrt(covar[v,v]) }

# log-likelihood

for (n in 1:N){

for (t in 1:T) { LL_task[n,t] <- log( prob[n,t, Y[n,t] ]) }

LL_resp[n] <- sum(LL_task[n,])

}

LL <- sum(LL_resp[])

# McFadden R-squared

LL_random <- N*T*log(0.5)

Rsq <- (LL - LL_random)/-LL_random

# QALY estimates (duration)

QALY_DUR[1] <- 1

for (v in 2:V){

QALY_DUR[v] <- mu_beta[v] / mu_beta[1]

}

#worst possible health state 55555

worst[1] <- QALY_DUR[1] + QALY_DUR[5] + QALY_DUR[9] +QALY_DUR[13] +QALY_DUR[17] + QALY_DUR[21]

# QALY estimates (immediate death)

QALY_DEAD[1] <- 1

for (v in 2:V-1){

QALY_DEAD[v] <- QALY_DUR[v] * (1/(1-QALY_DUR[V]) )

}

QALY_DEAD[V] <-0

worst[2] <- QALY_DEAD[1] + QALY_DEAD[5] + QALY_DEAD[9] +QALY_DEAD[13] +QALY_DEAD[17] + QALY_DEAD[21]

}

}
